# DIA proteomics identified the potential targets associated with angiogenesis in the mammary glands of dairy cows with hemorrhagic mastitis

**DOI:** 10.3389/fvets.2022.980963

**Published:** 2022-08-08

**Authors:** Quanwei Zhang, Xu Bai, Jun Shi, Xueying Wang, Bohao Zhang, Lijun Dai, Ting Lin, Yuan Gao, Yong Zhang, Xingxu Zhao

**Affiliations:** ^1^College of Veterinary Medicine, Gansu Agriculture University, Lanzhou, China; ^2^College of Life Science and Technology, Gansu Agriculture University, Lanzhou, China; ^3^Gansu Key Laboratory of Animal Reproductive Physiology and Reproductive Regulation, Lanzhou, China

**Keywords:** angiogenesis, CAV1, hemorrhagic mastitis, blood and cow, DIA proteomics

## Abstract

Hemorrhagic mastitis (HM) in dairy cows caused great economic losses in the dairy industry due to decreased milk production and increased costs associated with cattle management and treatment. However, the pathological and molecular mechanisms of HM are not well-understood. The present study aimed to investigate differentially expressed proteins (DEPs) associated with HM according to data-independent acquisition (DIA) proteomics. Compared to the mammary glands of healthylactating Holstein cows (Control, C group), the pathology of the HM group displayed massive alveolar infiltration of hemocytes and neutrophils, and the blood vessels, including arteriole, venules and capillaries were incomplete and damaged, with a loss of endothelial cells. DIA proteomics results showed that a total of 3,739 DEPs and 819 biological process terms were screened in the HM group. We focused on the blood, permeability of blood vessel, vascular and angiogenesis of mammary glands, and a total of 99 candidate DEPs, including 60 up- and 39 down-regulated DEPs, were obtained from the Gene Ontology (GO) and Pathway enrichment analyses. Phenotype prediction and function analysis of the DEPs revealed that three DEPs, particularly Caveolin-1(CAV1), were participated in the regulation of angiogenesis. Immunohistochemical and immunofluorescence staining showed that the CAV1 protein was present mainly in the mammary epithelial cells, vascular endothelial cells and vascular smooth muscle cells. The expression level of *CAV1* mRNA and protein in the HM group was significantly down-regulated. The results will be helpful to the further understanding of the pathological and molecular mechanisms of HM in dairy cows.

## Introduction

Mastitis in cows is categorized as a subclinical and clinical inflammatory disease of the mammary gland caused by a variety of stimulating factors, such as pathogens, physical and chemical factors and mechanical stimulus. It negatively affects animal welfare, milk quality, farmer serenity, and farming profitability and causes substantial economic losses throughout the world (1, 2). Previous researches have included investigation into the prevention and treatment of cow mastitis, such as vaccine development (2, 3), discovery of new drugs and new approaches of treatment (4), and exploring the veterinary medicinal compounds and monomer extracts of traditional Chinese medicine (5). Although, these have already yielded some significant progress, dairy mastitis remains a serious problem that prevents the healthy development of dairy industry around the world. The major shortcoming of previous studies is that the pathogenesis of mastitis has not been fully elucidated at the cellular, molecular and genetic levels, especially in cases of hemorrhagic mastitis.

Hemorrhagic mastitis, a form of clinical mastitis, is characterized by a dramatically reduced production of milk and the presence of pink floc or blood in the milk of dairy cows and usually occurs in high-yielding cows. Many undesirable exogenous and endogenous factors act on the mammary glands and can cause the blood vessels of the lactiferous ducts, alveoli and surrounding tissues to rupture and hemorrhage. For example, the mammary glands can be injured by blunt trauma, such as stomping, bruising and contusion. These injuries can be avoided by strengthening the feeding and management of dairy cattle and improving the safety and protection of barn conditions. In other cases, excess nutrients, high blood pressure and infectious pathogens can increase the permeability of blood vessels and allow the blood cells to seep from the blood vessel walls into the mammary gland, which also causes hemorrhagic mastitis. In a typical example, hemolysin-β from *Staphylococcus aureus* (*S. aureus*) is an important pathogenic factor in cattle that can promote the adhesion of *S. aureus* to bovine mammary epithelial cells (6) and release lysozymes to destroy the nearby tissues and cells, such as alveolar or vascular endothelial cells (7), which could cause clinical mastitis. It is difficult to control precisely the permeability of blood vessels, blood pressure, or the pathogenic process of infection, as these complex physiological processes are automatically regulated by the organism themselves. Therefore, it is necessary to identify the molecules related to hemorrhagic mastitis from the perspective of blood vessel permeability, angiogenesis and vascular repair. However, studies focusing on the pathogenesis of hemorrhagic mastitis and the associated candidate molecules (genes and proteins) remain rare. Previous studies suggested that dysregulated angiogenesis contributes to the progression of a number of inflammatory diseases (8), including clinical mastitis in dairy cows. The regulators of angiogenesis have yielded several candidates, such as vascular endothelial growth factors (VEGF), fibroblast growth factors (FGFs), transforming growth factors (TGFs), hepatocyte growth factor (HGF), tumor necrosis factor alpha (TNF-α), angiogenin, and interleukin-8 (IL-8) (8). Some of these factors [e.g., IL-8, TNF, and TGF (9, 10)] had been demonstrated to be associated with the inflammatory response and the development of mastitis in animals. Moreover, VEGF is a mitogen for vascular endothelial cells derived from arteries, veins and lymphatics that can promote angiogenesis and induce confluent microvascular endothelial cells to invade collagen and form capillary like structures (11). VEGF has also been shown to play important roles in inflammation (12) and *Escherichia coli* induced mastitis (13). Nevertheless, the relationship between these factors and cow hemorrhagic mastitis is incompletely described, due to a lack of relevant studies.

The goal of current study was to identify systematically candidate differentially expressed proteins (DEPs) or target molecules associated with the permeability of blood vessels, angiogenesis and vascular repair in cow hemorrhagic mastitis using bioinformatics analysis of Data-independent acquisition (DIA) proteomics. The results will provide a comprehensive insight into hemorrhagic mastitis and clinical mastitis.

## Materials and methods

### Sample preparation

The Holstein cows in healthy lactation (Control group, C, *n* = 3) or with hemorrhagic mastitis (Experimental group, HM, *n* = 3) were selected from a commercial farm in Wu Zhong City, Ningxia Province, China. Somatic cell count (SCC) and other criteria were applied as described previously (14). HM diagnosis was performed via veterinary clinical examination and milk samples observation as described previously (13, 15, 16). The Holstein cows with healthy and HM were transferred to a slaughterhouse in Wuzhong County, where the fresh mammary glands were obtained and immediately stored in liquid nitrogen or fixed with 4% paraformaldehyde. This study was approved by the Ethics Committee of Gansu Agriculture University, Lanzhou, China (No. GSAU-AEW-2018-0128).

### Directly DIA sequence and data analysis

The procedures of directly DIA proteomics were performed as described previously (15) using Orbitrap Exploris 480 system (Thermo Fisher Scientific, MA, USA). Raw Data of DIA were blasted to reference genome (NCBI_GCF_002263795.1), and further processed and analyzed by Spectronaut 14 (Biognosys AG, Switzerland) with default parameters. *Q* value (FDR) cutoff on precursor and protein level was applied 1%. All selected precursors passing the filters are used for quantification. The average top three filtered peptides which passed the 1% *Q* value cutoff were used for proteins quantification. The significantly different expressed proteins (DEPs) were filtered if their absolute ratio of fold change >1.50 and *Q* value < 0.05 using R packages. Gene Ontology (GO) and Kyoto Encyclopedia of Genes and Genomes (KEGG) pathway analyses were performed for obtaining their functions according to the DEPs as described previously (17). The GO terms and KEGG pathways with *Q value* ≤ *0.05* were considered statistically significant. The DIA proteomic data was deposited in iProX/ProteomeXchange with accession number IPX0003382000/PXD028100. The present study focused on the DEPs from the GO terms and pathways combined to the phenotype of hemorrhagic mastitis (8). The heat maps, Volcano plots, bubble and Upset Venn diagrams were drawn using the R language and the online OmicShare tools (https://www.omicshare.com/tools/) (17, 18). Protein-protein interaction (PPI) networks of the candidate DEPs were constructed using STRING v 10.0 (19), Cytoscape 2.8.1, including Clue-go, and Ingenuity pathway analyses (IPA) (20, 21). The Mouse Genome Informatics (MGI) database (22) was used for predicting possible phenotypes and functions when the target alleles was a mutation.

### Hematoxylin and eosin staining

Fixed mammary glands were embedded into paraffin (Solarbio, Beijing, China) and cut into 5 μm thick sections using a microtome (Leica, Shanghai, China). The sections were deparaffinized in xylene and rehydrated in an ethanol gradient. Hematoxylin and eosin (H&E) staining was carried out as described previously (23). The images of alveoli and blood vessels in the mammary glands were captured using an Olympus BX53M microscope (Olympus, Tokyo, Japan). All staining assays were performed at least in triplicate.

### Immunochemical staining

The sections were deparaffinized in xylene and rehydrated in an ethyl alcohol gradient. Antigen retrieval was performed using citrate buffer (Solarbio, Beijing, China). Caveolin-1 (CAV1) protein was detected using a standard avidin-biotin-peroxidase complex method of the ABC staining system (Bioss, Beijing, China) according to the manufacturer's instructions (18, 24). The rabbit polyclonal anti-CAV1 antibody (Bioss, Beijing, China) was diluted to 1:300. The negative control was set for specificity of antibody (PBS instead of CAV1 antibody). The images were captured using an Olympus microscope. All immune-staining assays were performed at least in triplicate.

### Immunofluorescence staining

Immunofluorescent staining (IF) with CAV1, CD31 (a marker of vascular endothelial cells, Bioss, Beijing, China) and α-SMA (a marker of vascular smooth muscle, Bioss, Beijing, China) antibodies was performed for co-localization analysis of vasculature in mammary glands as previously described (25). The sections were labeled with different primer antibodies at various dilutions (CAV1, 1: 150, CD31, 1: 250 and α-SMA, 1: 200) and incubated with the appropriate secondary antibody (CY3 for α-SMA, FITC for CAV1, and CY5 for CD31, Bioss, Beijing, China) at a 1:300 dilution after incubation with the primary antibodies. Nuclei were counterstained with a 10 μg/mL DAPI for 5 min. Fluorescent signals and images were captured using Pannoramic DESK slice scanner system (3D HISTECH Co., Budapest, Hungary). All immune-staining assays were performed at least in triplicate.

### RNA isolation, cDNA synthesis and qPCR assays

Total RNA was extracted from the mammary glands using a FastPure RNA isolation kit (Vazyme, Nanjing, China), following the manufacturer's instructions, and used for cDNA synthesis. The RNA concentration was quantified on a NanoDrop-8000 (ThermoFisher Scientific, Waltham, MA, USA) and RNA integrity was assessed by denaturing formaldehyde agarose gel (1%) electrophoresis (Biowest Regular Agarose, Castropol, Spain). One microgram of total RNA was subjected to reverse transcription to single-stranded cDNA using a BioTeke Thermo RT Kit (Bioteke, Beijing, China). The reverse transcription PCR reaction was carried out as described previously (18, 24). Relative expression levels of *CAV1* and *GAPDH* mRNA in the mammary gland tissues were measured using qRT-PCR. qRT-PCR primers were designed using Premier 5.0 software (24) and were synthesized by Qinke Biotech Co. Ltd. (Shanxi, China). The primers used for qRT-PCR were as follows, *CAV1* forward primer GCCGTGTCTATTCCATCT, and reverse primer ATTTCTTTCTGCGTGTTG; *GAPDH* forward primer GGTCACCAGGGCTGCTTT, and reverse primer CTGTGCCGTTGAACTTGC. qRT-PCR was performed in a 20 μL reaction volume, using 2 μL cDNA template and SYBR premix Ex Taq™ II, on a LightCycler 96 Real-time PCR system (Roche, Switzerland) according to the manufacturer's instructions. PCR procedures and result calculations were carried out as described previously (18, 24). The relative expression level of *GAPDH* was considered as an endogenous control and the expression levels of *CAV1* and *GAPDH* in the group C were used as controls. The results were calculated using the 2^−ΔΔCT^ method (18). All PCR assays were performed at least in triplicate.

### Western blot

The relative expression levels of CAV1 and β-actin proteins in the mammary glands from the C and HM groups were examined via Western blot. Total protein was extracted from 100 mg of each tissue sample using RIPA (Solarbio, Beijing, China). The procedures were carried out as described previously (18, 24). Bands indicating bound protein were scanned and their optical densities were quantified using Image-Pro Plus 6.0 software (Media Cybernetics Co., Rockville, MD, USA). β-actin was used as an endogenous control. The expression level of CAV1 protein in the C group was used a control. All immunoblot assays were performed at least in triplicate.

### Statistical analysis

All data were presented as the mean ± SD, unless otherwise indicated. Statistical analysis was performed using SPSS version 21.0 (SPSS Inc., Chicago, IL, USA). The qRT-PCR and Western blot data were analyzed using the Student's *t*-test (between two groups) or one-way ANOVA analysis (within multiple groups). The graphs were drawn using Prism 5.0 (GraphPad Software Inc., San Diego, CA, USA). *P* < 0.05 was considered to be statistically significant.

## Results

### Histologic observation of the mammary gland in Holstein cow with clinical mastitis

**Figure 1:**
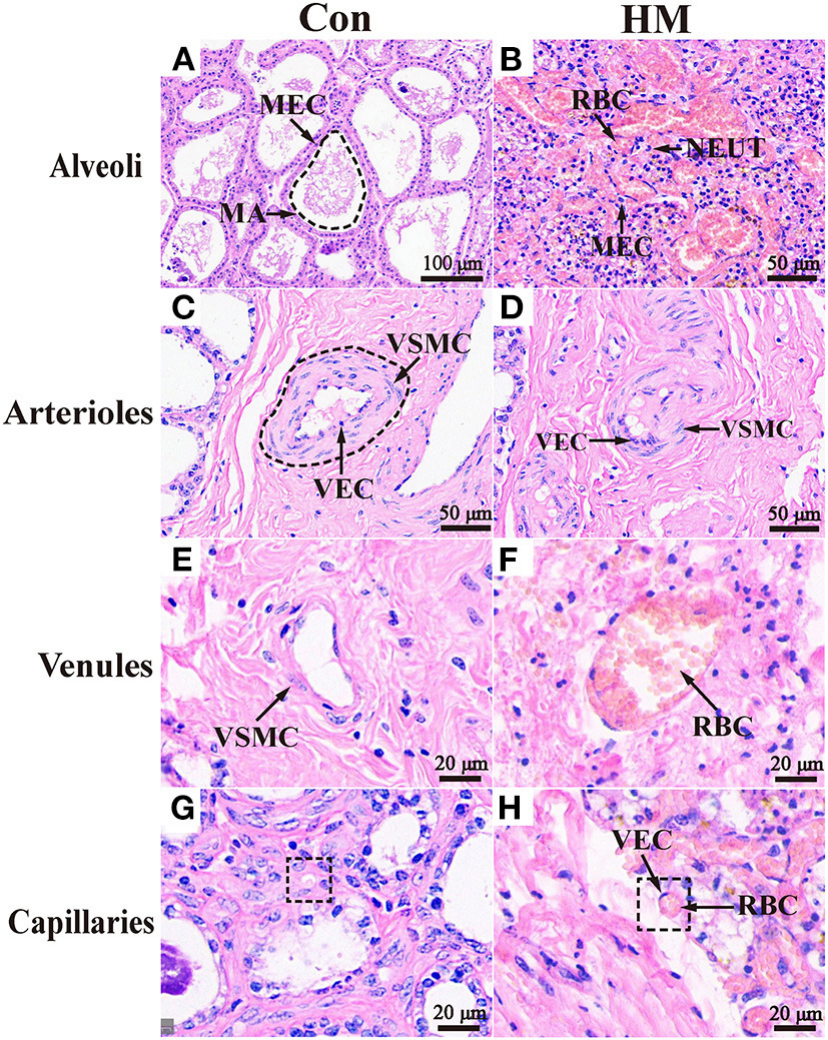
Pathological observation of the mammary glands in the Holstein cows. **(A,B)** Pathological variations of the alveoli in the mammary glands of the C and HM groups (100 ×). **(C–H)** Pathological variations of the arterioles **(C,D)**, venules **(E,F)**, and caillaries **(G,H)** in the mammary glands of the C and HM, respectively (100 ×). MEC, mammary epithelial cells; MA, mammary gland alveolar; RBC, red blood cell; NEUT, neutrophil; LY, lymphocytes; VEC, vascular endothelial cell; VSMC, vascular smooth muscle cell; Con, control group; HM, hemorrhagic mastitis group. Scale bar of 50, 100, and 20 μm represents 400×, 200 x, and 800 × magnification, respectively.

The mammary alveoli (MA) exhibited large alveolar luminal areas without evidence of inflammation, and the mammary epithelial cells (MECs) were intact and neatly arranged in the C group (Figure 1A). However, the MA of the mammary glands were infiltrated by massive hemocytes and neutrophils that could be observed in the secretions of the glandular ducts, alveoli and ducts with some exfoliated epithelial cells in the HM group (Figure 1B). The blood vessels in the connective tissue of the mammary glands were expanded and blood cells filled the vascular cavity. The cross-sections of the arterioles of the C group were regular with obviously elastic intima and undamaged vascular cavities (Figure 1C), whereas the arteriolar endothelium in the mammary glands of the HM group was detached and displayed collapsed lumen and damaged blood vessel walls (Figure 1D). The boundaries of the venules and blood vessels in the mammary glands of the group C were clearly visible with the smooth luminal endothelium (Figure 1E). However, the venular vascular endothelium in the mammary glands of the group HM were damaged with the endothelial cell detachment. The media and adventitia of vessels were separated (Figure 1F). The mammary gland capillaries of the group C were composed of a layer of endothelial cells and the morphological structure was complete and clear without inflammatory cells (Figure 1G). The capillaries were severely dilated and surrounded by massive inflammatory cells, such as neutrophils and lymphocytes, in the mammary glands of the group HM (Figure 1H).

### Quality evaluation and DEPs identification of DIA data

**Figure 2:**
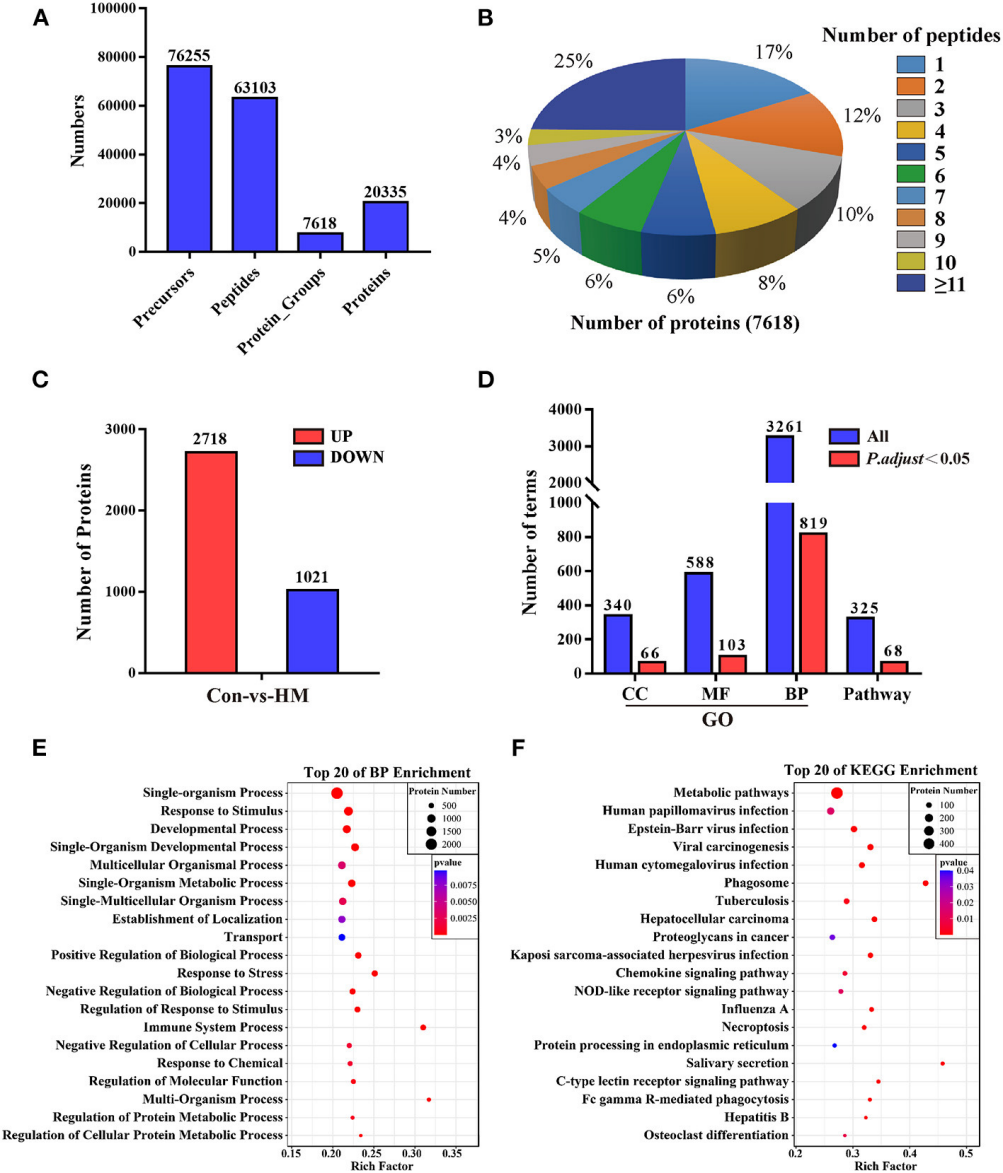
Summary of the proteome analysis, protein identification and annotation in DIA data. **(A)** statistics for the peptides and proteins identification. **(B)** peptides number distribution analysis. The percentage represents the proportion of the number of peptides constituting the proteins. **(C)** statistics for the DEPs compared to the C group. **(D)** statistics for the GO terms including BP, MF, and CC, and pathway annotation. BP, biological process; MF, molecular function; CC, cellular component. **(E,F)** The top 20 biological process in GO annotation and the top 20 pathways in pathway annotation, respectively. Con, control group; HM, hemorrhagic mastitis group; GO, gene Ontology terms.

A total of 76,255 precursors, 63,103 peptides, 7,618 protein groups and 20,355 proteins were identified with a filter criterion of FDR < 0.01, respectively (Figure 2A). The results of peptides number distribution analysis suggested that 25% proteins were constituted by 11 or more peptides, 17% proteins were constituted by one peptide, others were constituted by two to 10 peptides (Figure 2B). Compared to the C group, a total of 3,739 DEPs (Supplementary Table 1) including 2,718 up-regulated and 1,021 down-regulated DEPs were screened in the HM group (Figure 2C). These DEPs were applied for GO and pathway annotation, and the results showed that a total of 819 biological process terms (BP), 103 molecular function terms (MF), 66 cellular component terms (CC), and 68 pathways with *P* < *0.05* and *P. adjust*< *0.05* were identified, respectively (Figure 2D). Taken BP for example, most of the BPs were related to metabolic processes, response to stimulus and stress, immune system processes (Figure 2E). Most of the pathways were associated with infection, immunization and inflammatory diseases (Figure 2F).

### Identification of candidate DEPs related to angiogenesis from the GO terms

**Figure 3:**
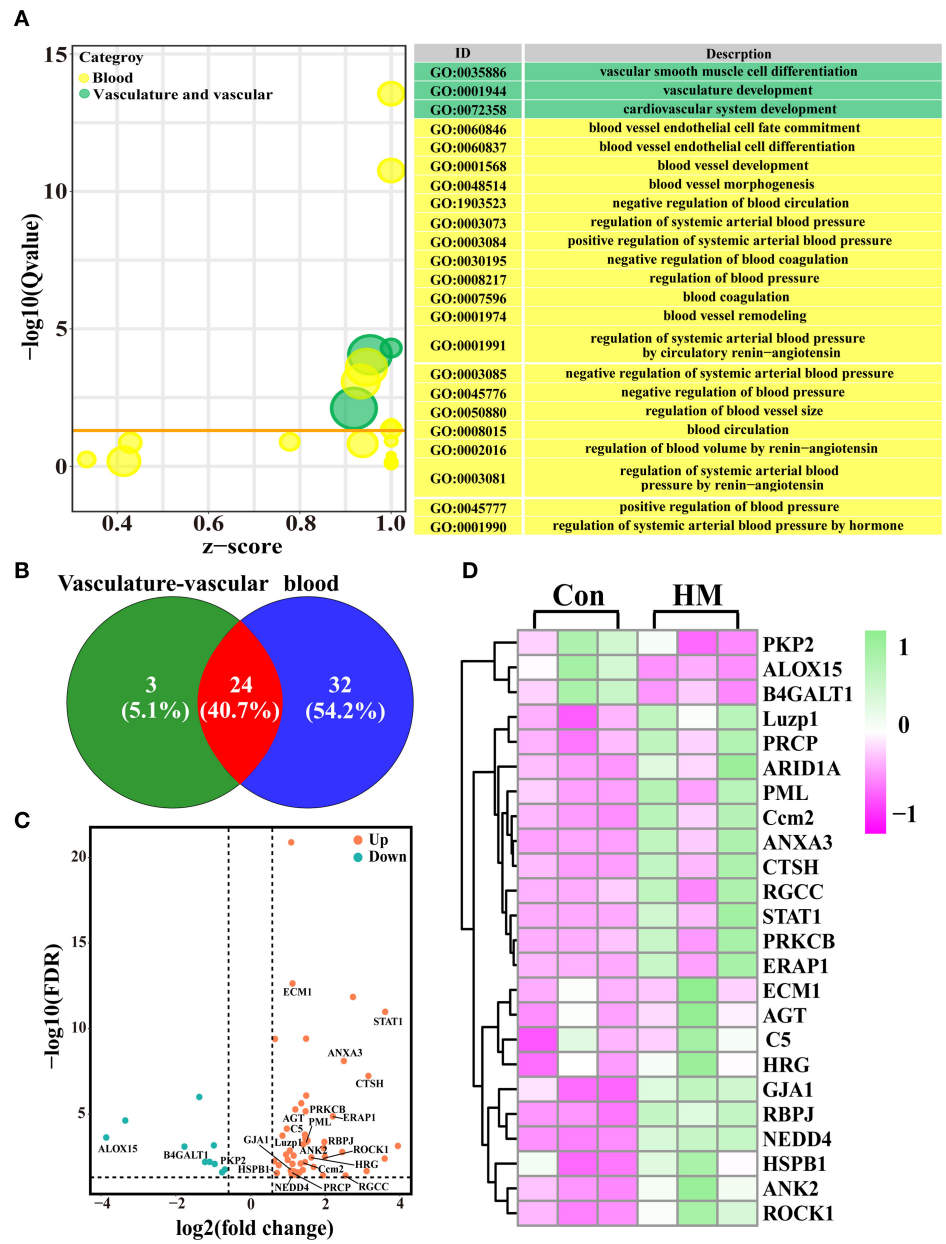
The candidate DEPs selected from the 3,739 DEPs and GO terms related to angiogenesis. **(A)** the GO terms selected from the 819 significantly different biological processes related to blood, vascular or vasculature, and angiogenesis in the DIA data. **(B)** Venn diagram of the 59 DEPs related to blood and vasculature or vascular development. **(C)** Volcano plots of the 24 co-expressed DEPs. **(D)** Heat-map of the representative 24 co-expressed DEPs. Con, control group; HM, hemorrhagic mastitis group.

A total of 27 DEPs included in three GO terms related to cardiovascular system development, vascular smooth muscle cell differentiation and vasculature were selected. A total of 56 DEPs included in 20 GO terms (e.g., blood circulation, blood vessel remodeling and regulation of systemic arterial blood pressure) related to blood were selected for further study (Figure 3A). After accounting for overlapping DEPs, a total of 59 DEPs (Supplementary Table 2) and 24 co-expressed DEPs were identified as candidate DEPs that might affect the function and variation of blood, vasculature or vascular development and angiogenesis based on the Venn diagram (Figure 3B). Volcano plots were created to observe the expression levels, and the results showed that 49 of the 59 DEPs were up-regulated, while 10 of the 59 DEPs were down-regulated (Figure 3C). A heat-map of the representative DEPs and 24 co-expressed DEPs was constructed to analyze the expression differences both in the C and HMgroups. The results showed that, between the C and HM groups, these DEPs were significantly differentially expressed in the mammary glands (Figure 3D).

### PPI network of the candidate DEPs selected from the GO terms

**Figure 4:**
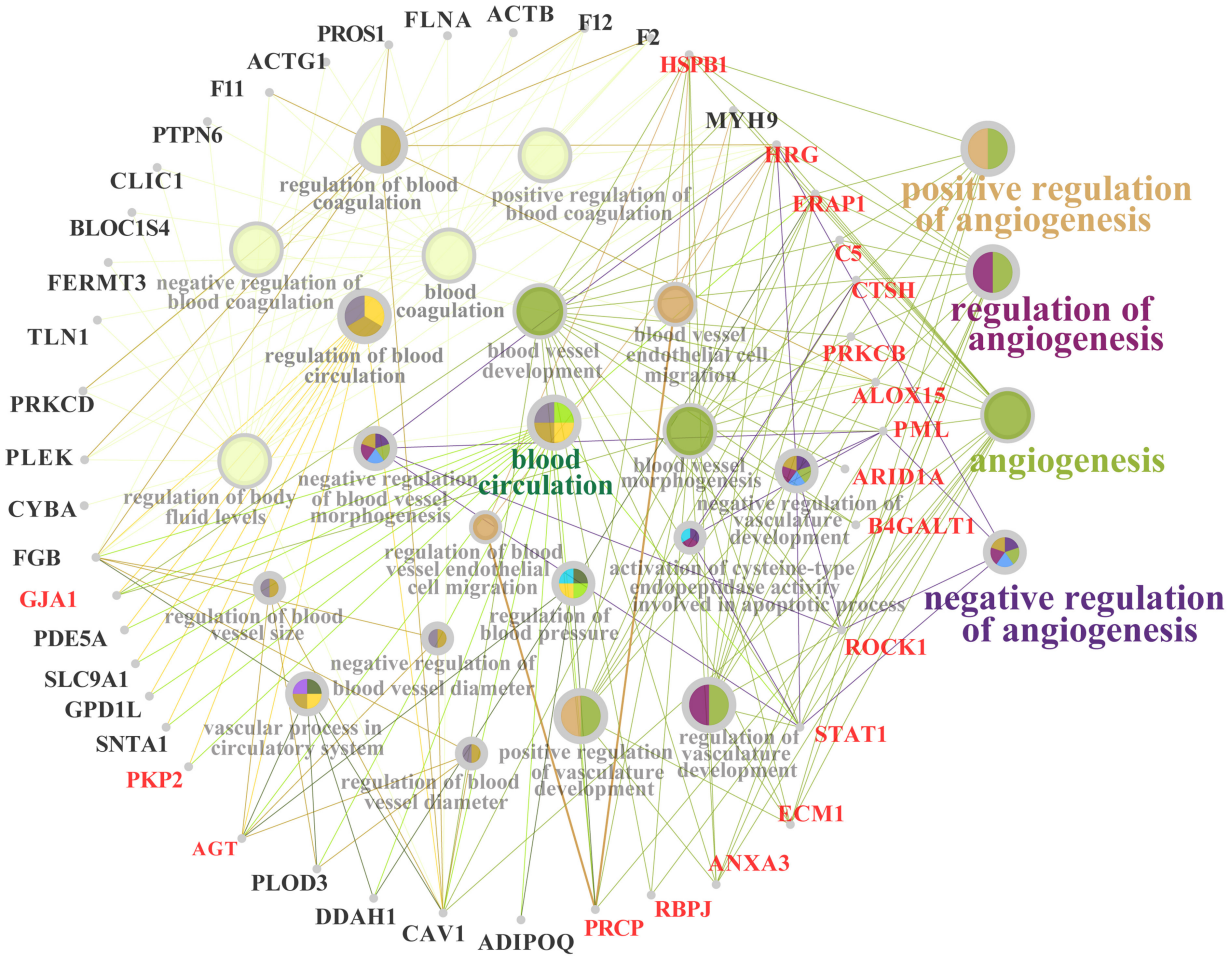
PPI network of the 59 candidate DEPs and 25 GO terms related to blood, vascular and angiogenesis.

A PPI network of the 59 candidate DEPs and the participating GO terms was constructed to understand further the functions and regulatory roles in protein-protein and protein-GO term interactions (Figure 4). A total of 25 GO terms, including blood circulation, regulation of blood pressure and regulation of blood vessel diameter, which are crucial biological processes impacting animal metabolism and homeostasis, interacted with the 44 DEPs. These DEPs were directly or indirectly involved in regulation of the 25 GO terms. For example, HGR, ERAP1, HSPB1, and STAT1 were involved in 10 or more GO terms. Interestingly, most of the DEPs or GO terms directly participated in four important angiogenesis related GO terms, such as positive regulation of angiogenesis, regulation of angiogenesis and negative regulation of angiogenesis. The results suggested that these DEPs may be responsible for regulation of blood or the blood circulation system.

### Identification of candidate DEPs related to blood and vascular from the KEGG pathways

**Figure 5:**
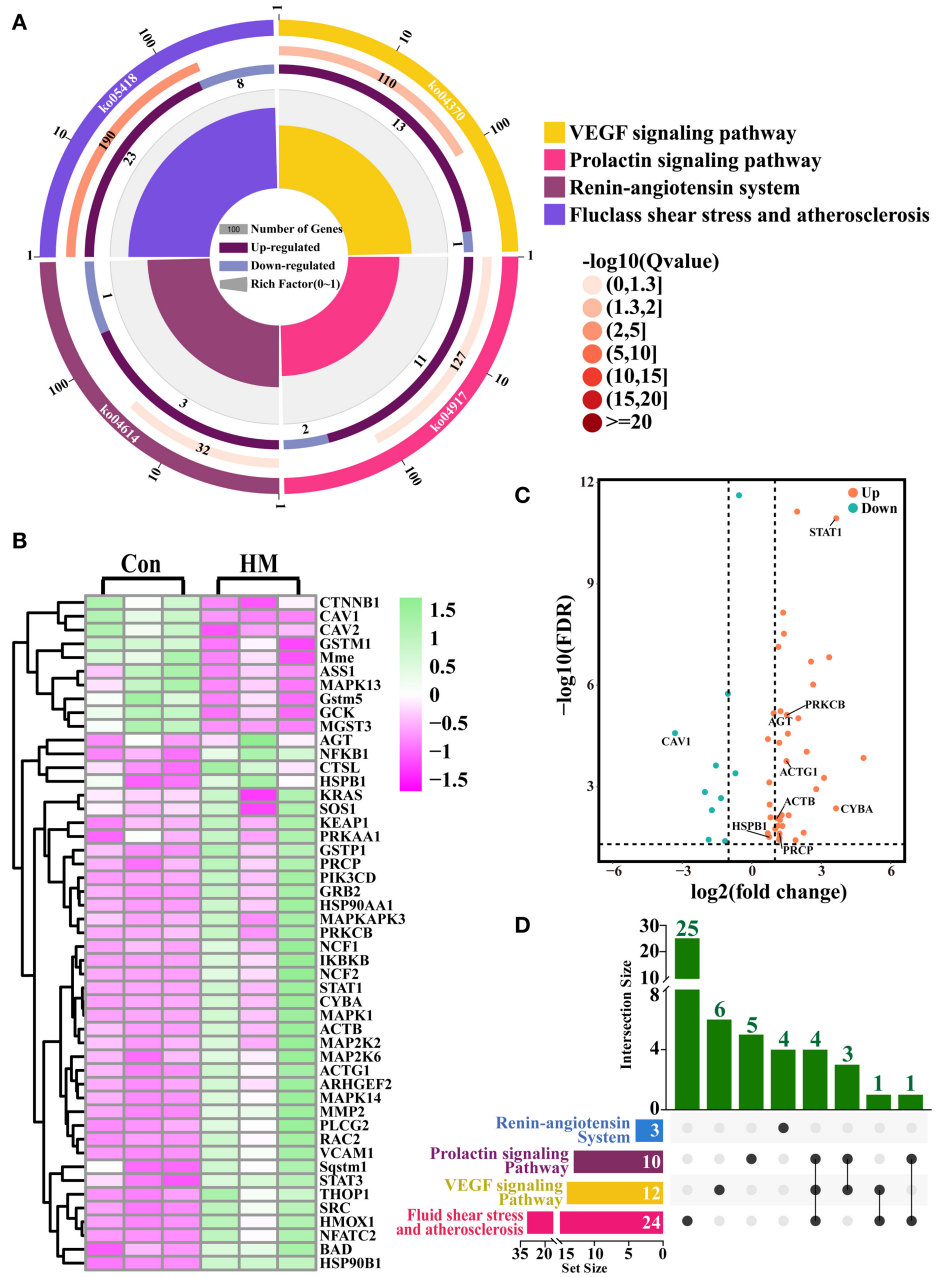
The candidate DEPs and pathways related to blood, vascular or vasculature, and angiogenesis. **(A)** the candidate pathways related to blood, vascular or vasculature, and angiogenesis selected from the 68 significantly different pathways according to KEGG annotation of the 3,739 DEPs. **(B)** heat-map of the 49 DEPs selected from the four significantly different pathways. **(C)** Volcano plots of the 49 DEPs including 10 down-regulated and 39 up-regulated proteins. **(D)** Upset Venn diagram of the 49 DEPs and the four pathways. Con, control group; HM, hemorrhagic mastitis group.

Four significantly different pathways, VEGF signaling pathway, prolactin signaling pathway, renin-angiotensin system, and fluid shear stress and atherosclerosis, included 49 DEPs (Supplementary Table 3) that were chosen for further study (Figure 5A). The constructed heat-map showed that these DEPs were significantly differentially expressed in the mammary glands of the C group compared to the HM group (Figure 5B). The Volcano plots showed that ten of the 49 DEPs, including CAV1, were down-regulated while 39 of the 49 DEPs, such as STAT1, AGT, and PRKCB, were up-regulated (Figure 5C). The upset Venn diagram suggested that no DEPs were co-expressed in these four pathways. Most of these DEPs were presented in one pathway. Only MAPK13 and MAPK14, SRC, and PIK3CD were expressed in two or three pathways (Figure 5D).

### Functional analysis of the target DEPs

**Figure 6:**
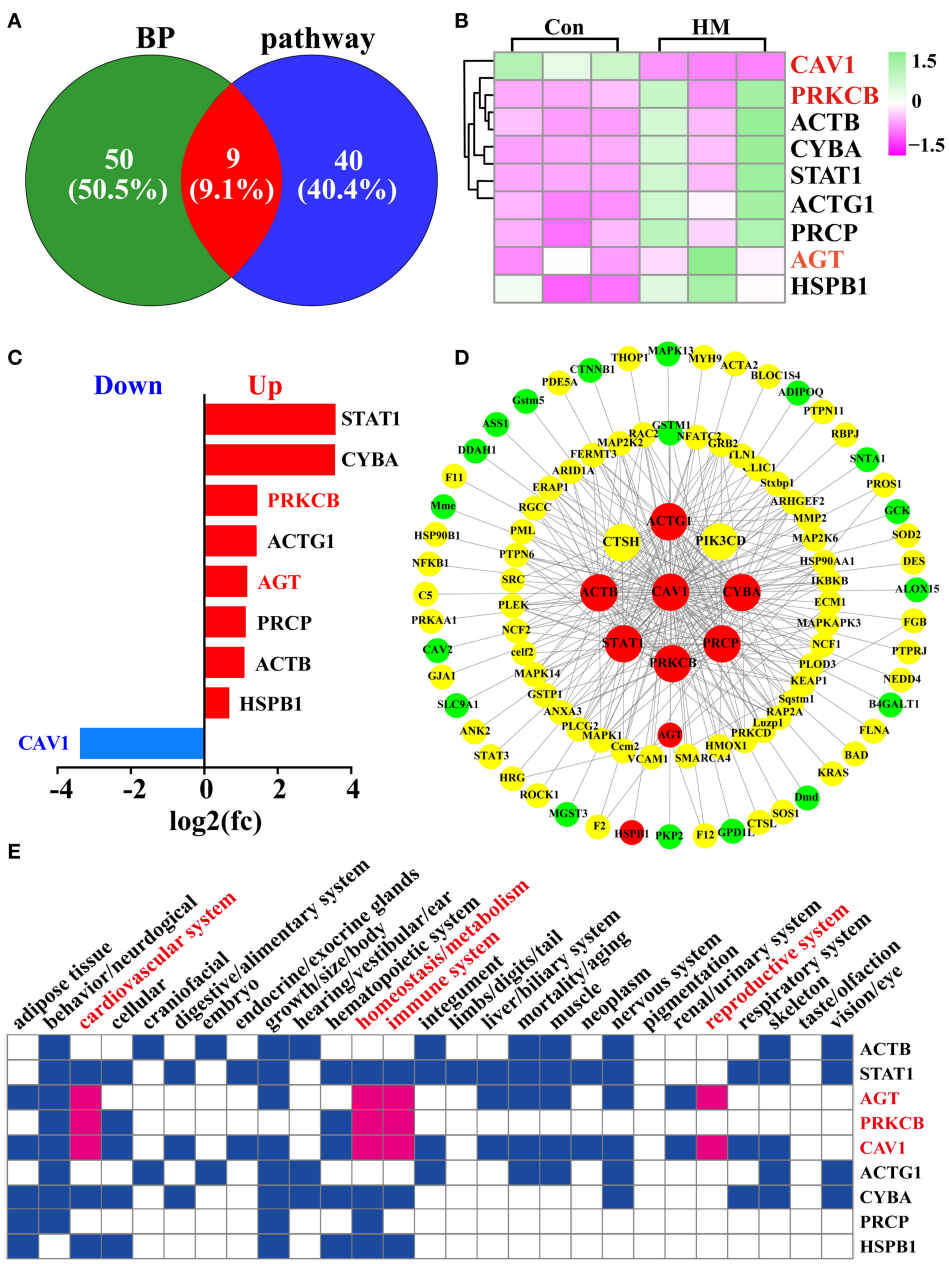
Identification and functional analysis of DEPs related to blood, vascular or vasculature, and angiogenesis selected from the GO terms and KEGG pathways. **(A)** Venn diagram of the DEPs related to blood, and vasculature or vascular selected from the GO terms and KEGG pathways. **(B)** Heat-map of the nine shared DEPs selected from the GO terms and KEGG pathways. **(C)** the relative expression levels of the nine shared DEPs quantified by DIA proteomics using log_2_ (FC) values. **(D)** PPI network of the 99 DEPs after overlapping GO terms and KEGG pathways. **(E)** phenotype prediction and functional analysis of the nine representative DEPs. Con, Control group; HM, Hemorrhagic mastitis.

Candidate DEPs selected from the GO terms and KEGG pathways were obtained for further function analysis. The Venn diagram was constructed, and the results showed a total of 99 DEPs, including 50 DEPs unique in the GO term biological processes, 40 DEPs unique to KEGG pathways and nine co-expressed DEPs both in biological processes and KEGG pathways (Figure 6A). The heat-map of the nine shared DEPs was established and the results indicated that these DEPs were significantly differentially expressed between the C and HM groups (Figure 6B). The relative expression levels of these shared DEPs were quantified using log_2_ (FC) values and the results showed that there was one down-regulated and eight up-regulated DEPs (Figure 6C). Meanwhile, using these DEPs as the core, the interaction network of the 99 DEPs was constructed and the results showed that 43 of the 99 DEPs interacted with only one DEP, five interacted with two DEPs, and 51 of the 99 DEPs interacted with two or more DEPs (Figure 6D). The Mouse Genome Informatics (MGI) database was used for phenotype prediction and functional analysis of some representative DEPs (Supplementary Table 4), and the results suggested that six DEPs were correlated with some important biological processes, such as cardiovascular system, in particular AGT, PRKCB and CAV1. These three DEPs were also related to some important biological processes associated with mammary glands, such as immune system and reproduction system (Figure 6E). For example, in a mutated CAV1 allele, the mice displayed vascular system dysfunctions and lung alveolar septa showing hyper proliferation and fibrosis.

### Validation of *CAV1* mRNA and protein

**Figure 7:**
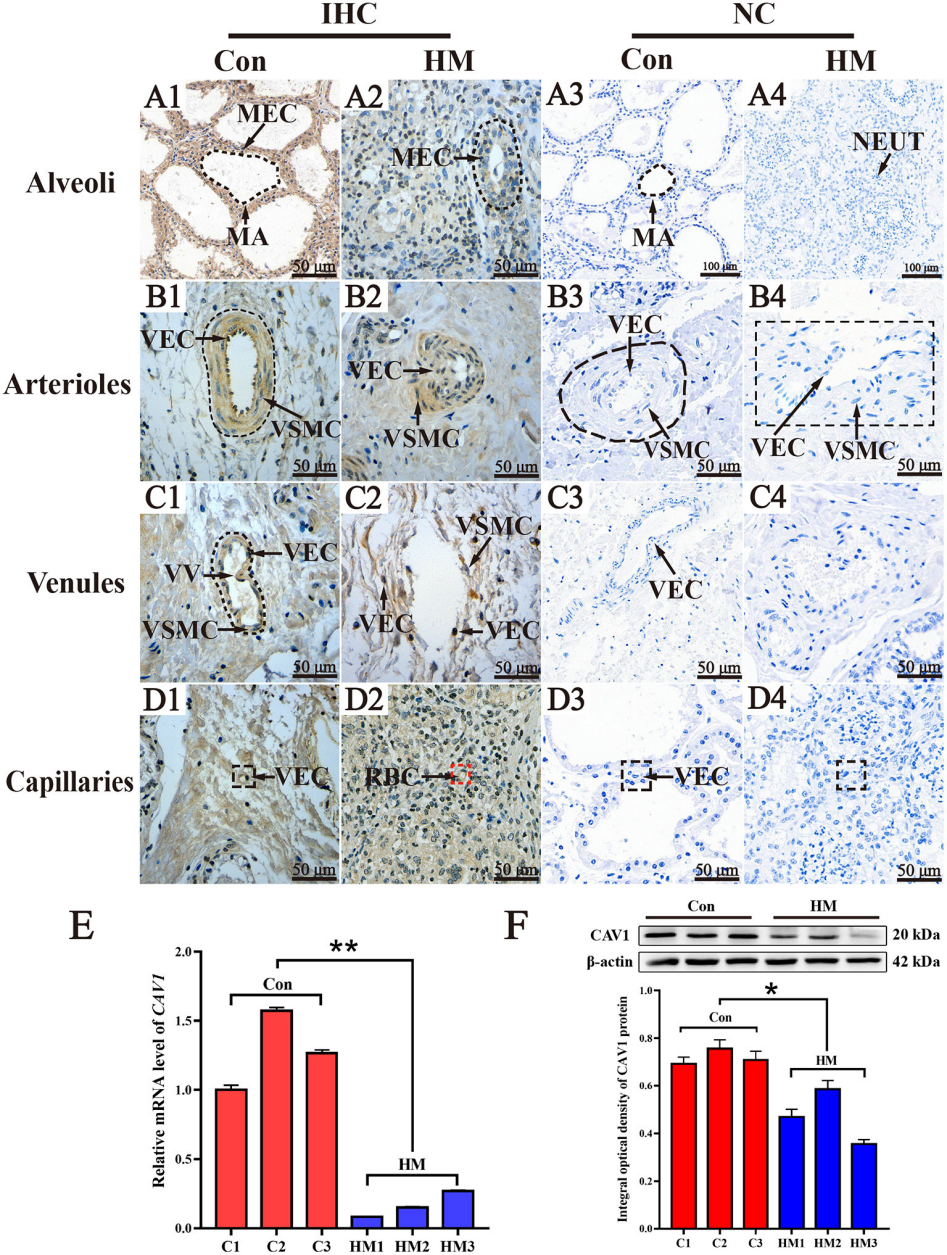
The subcellular location, mRNA and protein analysis of CAV1 in the mammary glands of healthy Holstein cows with hemorrhagic mastitis. **(A1–D1,A2–D2)** the subcellular location of CAV1 protein in the alveolus, arteriole venules and capillaries of the mammary glands in the C and HM groups, respectively. **(A3–D3,A4–D4)** the negative control mammary glands of Holstein cow, respectively. **(E,F)** the relative expression of *CAV1* mRNA and protein in the C and HM groups, respectively. MEC, mammary epithelial cells; MA, mammary gland alveolus; VEC, vascular endothelial cell; VSMC, vascular smooth muscle cell; NEUT, neutrophil; VV, venous valve; Con or C, control group, the mammary gland of healthy Holstein cow; HM, hemorrhagic mastitis group, the mammary gland of Holstein cow with hemorrhagic mastitis. Scale bar of 50 and 20 μm represents 400 × and 800 × magnification, respectively. *, significant difference; **, extremely significant difference.

CAV1 was selected as one of the target DEPs for validating. The results of IHC staining showed that CAV1 protein was present in the MECs, vascular endothelial cells (VECs) and vascular smooth muscle cells (VSMCs). In the MA, CAV1 was observed both in the cytoplasm and nucleus of MECs with differential staining degrees between the C and HM groups. Generally, the immuno-positive reaction of CAV1 protein in the C group was higher than that in the HM group (Figures 7A1,A2). Compared to the HM group, the strong positive detection of CAV1 protein was also seen in the cytoplasm and nucleus of arteriolar VECs and VSMCs in the mammary glands of the C group (Figures 7B1,B2). The strong CAV1 protein detection was seen in the cytoplasm and nucleus of venous VECs and SMC in the mammary glands of the C group. Conversely, CAV1 protein was detected only weakly in the exfoliated VECs and VE in the mammary glands of the HM group (Figures 7C1,C2). In mammary gland capillaries, CAV1 protein was strongly stained in the cytoplasm and nucleus of VECs in both the C and HM groups (Figures 7D1,D2) CAV1 protein was not seen in the negative control and was not present in mammary gland tissues (Figures 7A3,A4,B3,B4,C3,C4,D3,D4). Compared to the C group, the expression level of *CAV1* mRNA in the HM group was significantly down-regulated (*P* < 0.05, Figure 7E). Meanwhile, the relative expression level of CAV1 protein in the mammary glands of the C group was significantly higher than that in the HM group (*P* < 0.05, Figure 7F).

### IF localization analysis of CAV1 protein in the mammary gland tissues

**Figure 8:**
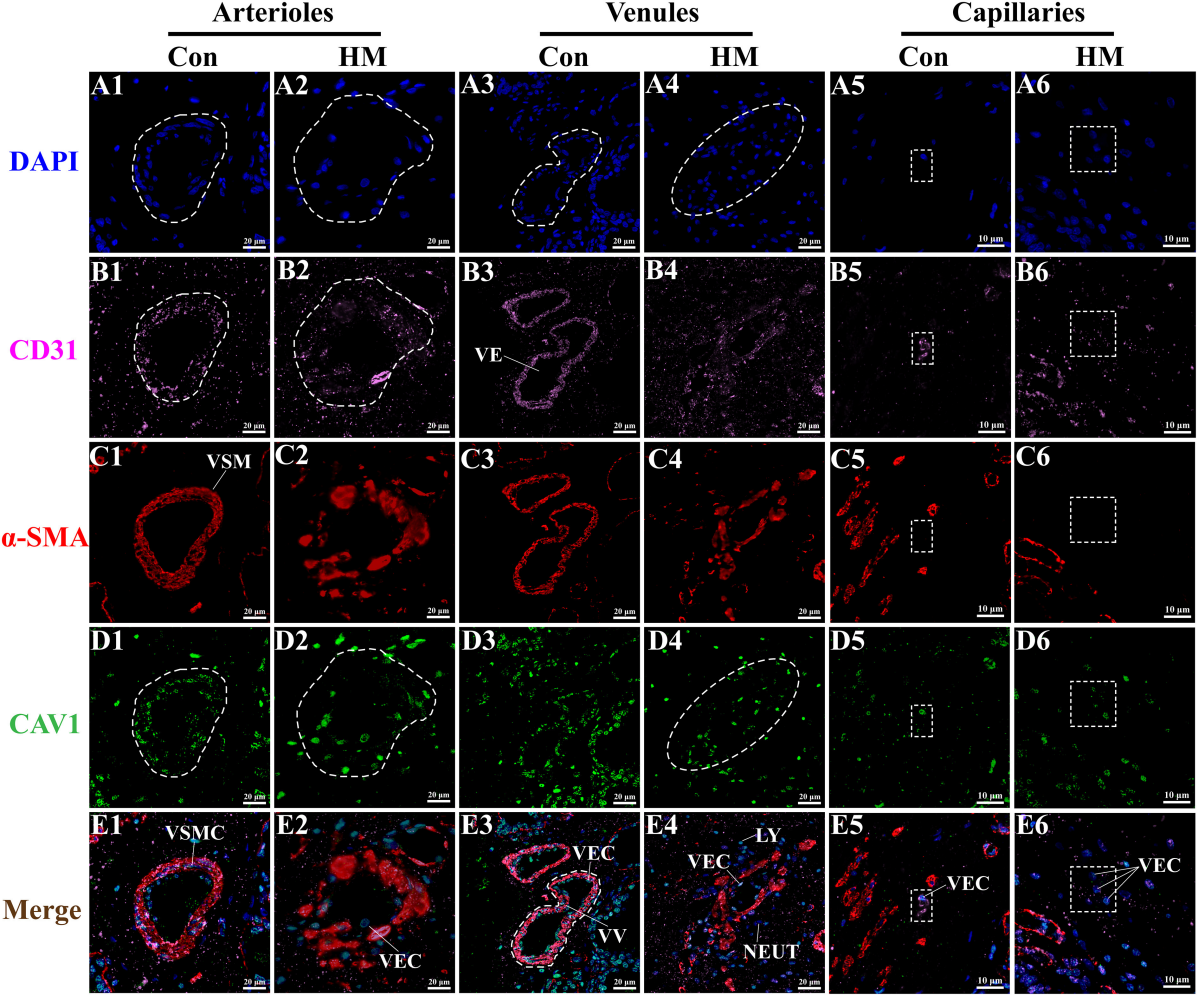
Co-localization analysis, via immunofluorescent staining, of CAV1 protein in the mammary gland tissues of healthy and hemorrhagic mastitis Holstein cows. **(A1–A6)** the nuclei of different types of cells labeled with DAPI. **(B1–B6)** CD31 protein in the VECs of blood vessels. **(C1–C6)** α-SMA protein in VSMCs of blood vessels. **(D1–D6)** localization analysis of CAV1 protein in the mammary gland tissues. **(E1–E6)**, Co-localization analysis of CAV1, CD31, and α-SMA proteins in blood vessels of the mammary gland tissues in healthy and experimental Holstein cows. VEC, vascular endothelial cell; VSMC, vascular smooth muscle cell; NEUT, neutrophil; VV, venous valve; Con or C, Control group; HM, Hemorrhagic mastitis group. Scale bar of 50 and 20 μm represents 400 × and 800 × magnification, respectively.

The nuclei of different types of cells, including the VECs and VSMCs, labeled with DAPI were clearly observed in the mammary gland tissues in both the C and HM groups (Figures 8A1–A6). The IF signals of CD31 protein were found mainly in the cytoplasm of VECs (Figures 8B1–B6), particularly in the arterioles of the C group (Figure 8B3). α-SMA protein signals were found primarily in the cytoplasm of VSMCs in the alveoli and arterioles of the mammary gland tissues in the C and HM groups (Figures 8C1–C6). The alveoli, arterioles and capillaries of the C group were clear and complete, based on the IF signals of CD31 and α-SMA proteins. However, the alveoli, arterioles and capillaries of the mammary gland tissues in the HM group were incomplete and the VECs were detached. Localization analysis of CAV1 in the mammary glands showed that the CAV1 protein was exhibited mainly in the cytoplasm and nucleus of VECs with differential staining degrees in the arterioles of the C group (Figures 8D1–D6). Combining these signals, the basic morphology of the blood vessels was formed and further used for localization analysis (Figures 8E1–E6). The results revealed that the morphology of alveoli, arterioles and capillaries in the mammary gland tissues of the C group were intact and the VECs and SMCs were neatly arranged (Figures 8E1,E3,E5). However, the morphology of alveoli, arterioles and capillaries of the HM group were damaged, with detached VECs and increased vascular permeability (Figures 8E2,E4,E6), consistent with the results of H&E and IHC staining.

## Discussion

Homeostasis is crucial for the balance of metabolism, immune regulation and circulatory systems in animals. This biological process depends mainly on the exchange of substances between the blood and tissue fluids. Blood vessel permeability is essential for homeostasis and substance exchange and is highly variable as a result of intrinsic and extrinsic factors (26), particularly in cases of tissue inflammation. HM, as a result of increased inflammation of the mammary glands, is characterized by the presence of blood clots in milk and hemocytes in the MA (27). Generally, hemocytes, as macromolecular substances, are not allowed to filter into tissue fluid or interstitial fluid unless blood vessels are damaged or these exists an increase of blood vessel permeability (28). However, the correlation between these phenomena and HM remains unclear. In the present study, we have focused on variations of blood vessel permeability and associated candidate proteins in the mammary glands from lactating Holstein cows with HM.

H&E staining results suggested that the inflammatory cells were presented in the mammary glands, which might increase the inflammatory response and damaged blood vessels. Many cytokines secreted by inflammatory cells (29) and MECs can increase blood-brain barrier permeability and blood vessel permeability by modifying intracellular signal transduction directly or via receptors (30). Subsequently, we focused on blood vessels in the mammary glands and the results revealed that the blood vessels displayed more substantial hemangiectasis, abscission of VECs, damage to the blood vessel wall and separation of the media and adventitia compared to that seen in the C group. These variations are the typical characteristics of blood vessel injury or permeability changes (31). Increased microvascular permeability during angiogenesis possibly facilitates the extravasation of proteins (31), cells and pathogens. Therefore, these results demonstrated that blood vessel injury or changes in permeability in the mammary glands have a close relationship with the occurrence and development of HM.

In order to investigate the relevant mechanism of HM, DIA proteomics was used to identify the DEPs related to angiogenesis in mammary glands. Compared to the C, a total of 3,739 DEPs were identified in the HM group, which were more than that in previous studies (14). Some of the DEPs such as TLR2, STAT1 and Cathelicidins were associated with inflammation of dairy mastitis (32–34). According to the pathological characteristics in the mammary glands, the DEPs from GO terms and KEGG pathways related to blood vessels and blood vessel permeability were selected for the current study. The results showed that a total of 99 DEPs, including 59 DEPs in 25 GO terms and 49 DEPs in four KEGG pathways, were associated with angiogenesis. Previous studies have already shown that these biological processes are highly correlated with angiogenesis (8, 11, 26, 29). For example, several studies have shown that VEGF signaling can mediate vascular permeability by activating MMP9 (35) and can regulate edema and vessel injury (36). Meanwhile, some candidate DEPs, such as AGT (37), HSP1 (38), and STAT1 (39), have also been shown to mediate angiogenesis via the VEGF signaling pathway in different physiological processes. The IPP and IPA networks revealed that most of the DEPs did not participate directly in regulation of angiogenesis. However, they can mediate angiogenesis by binding to other receptors or proteins related to angiogenesis. Some DEPs, such as SNTA1, SLC9A1, and FERMT3, lack relevant evidence of participation in angiogenesis regulation. However, previous studies have described that these DEPs play important roles in cell migration and apoptosis (40), breast cancer and inflammation (41) processes that are also highly related to angiogenesis (42). This indicates that the functions of these DEPs require further study. The phenotypes prediction and function analysis displayed that some DEPs could directly affect angiogenesis, while the others were not involved in angiogenesis.

These DEPs likely affected angiogenesis through other pathways such as ACTB, PRCP and ACTG1, which were probably related to animal growth. However, the formation of new vessels also depends on interactions between various hormones and relevant growth factors (43). Thus, these DEPs could affect angiogenesis via regulation of growth factors.

CAV1, as a representative DEP related to angiogenesis was selected for verification. The results indicated that CAV1 might be play an important role in VECs and MECs in the mammary glands. CAV1 is a protein partner of endothelial nitric oxide synthase (eNOS) that is expressed in vascular endothelial cells and negatively regulates the activity of eNOS in vascular tone, platelet aggregation and angiogenesis (44). Previous research described that CAV1 is associated with the vascular invasion and regional lymph node metastasis in canine mammary tumors (44). Collectively, our results highlighted some DEPs associated with angiogenesis in the mammary glands of dairy cows with HM. However, the molecular mechanism of these DEPs in regulation of angiogenesis and HM remains incompletely elucidated and requires further study.

## Conclusions

In the present study, we found that hemorrhagic mastitis in Holstein cows was associated with pathologic variations of alveoli and blood vessels in the mammary gland. Compared to the C group, a total of 3,739 DEPs and 819 biological process terms were identified in the HM group according to the DIA data, respectively. Among these, a total of 99 target DEPs related to blood, vascular or vasculature, and angiogenesis were obtained. Functional analysis revealed that these DEP were participated in regulation of angiogenesis in the mammary glands, especially CAV1. Verification results suggested that CAV1 protein was present mainly in the cytoplasm and nucleus of MECs, VECs and SMCs. The expression levels of *CAV1* mRNA and protein in the HM group were significantly down regulated. These results indicated that these DEPs are the targets associated with angiogenesis in the mammary glands in dairy cows with hemorrhagic mastitis.

## Data availability statement

The datasets presented in this study can be found in online repositories. The names of the repository/repositories and accession number(s) can be found in the article/Supplementary material.

## Ethics statement

The animal study was reviewed and approved by Local Ethics Committee of the College of Veterinary Medicine, Gansu Agriculture University, Lanzhou, China (approval number GSAU-AEW-2018-0128).

## Author contributions

QZ: conceptualization. XB: methodology. QZ, BZ, XB, and JS: software. QZ, XB, JS, LD, TL, and XW: validation and data curation. QZ, XB, YG, and JS: formal analysis. XB, JS, LD, TL, and XW: investigation. QZ, XB, and JS: writing—original draft preparation. QZ and XZ: writing—review and editing and visualization. QZ, YZ, and XZ: supervision, project administration, resources, and funding acquisition. All authors have read and agreed to the published version of the manuscript.

## Funding

This study was supported by the Subject Construction Funding of Gansu Agricultural University, Gansu Province, China (GAU-XKJS-2018-160), the National Natural Science Foundation of China (No. U21A20262), the Funding of Gansu Key Laboratory of Animal Reproductive Physiology and Reproductive Regulation Gansu Province, China (GAU-XKJS-2018-160), and the Improvement Project of Higher Education Department of Gansu Province, China (2019A-049). These funding bodies had no role in the design of the study, the collection, analysis, and interpretation of the data, or the writing of the manuscript.

## Conflict of interest

The authors declare that the research was conducted in the absence of any commercial or financial relationships that could be construed as a potential conflict of interest.

## Publisher's note

All claims expressed in this article are solely those of the authors and do not necessarily represent those of their affiliated organizations, or those of the publisher, the editors and the reviewers. Any product that may be evaluated in this article, or claim that may be made by its manufacturer, is not guaranteed or endorsed by the publisher.
